# Expression Profile Analysis of Differentially Expressed Circular RNAs in Steroid-Induced Osteonecrosis of the Femoral Head

**DOI:** 10.1155/2019/8759642

**Published:** 2019-11-15

**Authors:** Zhongxin Zhu, Wenxi Du, Huan Yu, Hongting Jin, Peijian Tong

**Affiliations:** ^1^Zhejiang Chinese Medical University, Hangzhou, Zhejiang 310053, China; ^2^Institute of Orthopaedics and Traumatology of Zhejiang Province, Hangzhou, Zhejiang 310053, China; ^3^Department of Orthopaedic Surgery, The First Affiliated Hospital of Zhejiang Chinese Medical University, Hangzhou, Zhejiang 310053, China

## Abstract

**Background:**

A growing number of studies have suggested that circular RNAs (circRNAs) serve as potential diagnostic biomarkers in many diseases. However, the role of circRNAs in steroid-induced osteonecrosis of the femoral head (SONFH) has not been reported.

**Methods:**

Secondary sequencing was performed to profile circRNA expression in peripheral blood samples from three SONFH patients and three healthy individuals. We confirmed our preliminary findings by qRT-PCR. Bioinformatics analysis was conducted to predict their functions.

**Results:**

The result showed 345 dysregulated circRNAs. qRT-PCR of eight selected circRNAs preliminarily confirmed the results, which were consistent with RNA sequencing. Bioinformatics analyses were performed to predict the functions of circRNAs to target the genes of miRNAs and the networks of circRNA-miRNA-mRNA interactions.

**Conclusions:**

This study provides a new and fundamental circRNA profile of SONFH and a theoretical basis for further studies on the functions of circRNAs in SONFH.

## 1. Background

Steroid-induced osteonecrosis of the femoral head (SONFH) is an intractable disorder caused by the use of glucocorticoids (GCs) and leads to the progressive necrosis of osteocytes and the bone marrow [1, 2]. SONFH cases are increasing owing to long-term GC use or high-doses of GCs in patients [3]. Patients with SONFH are commonly asymptomatic, which makes its early diagnosis a challenge. SONFH usually occurs within two years after GC treatment, typically within the first six months [4]. Various joint preservation procedures have been performed for the treatment of precollapse disease [5]. However, in the absence of appropriate therapeutic interventions in the early stage, the collapse of the femoral head may later become unavoidable, resulting in severe secondary osteoarthritis and warranting artificial joint replacement in nearly 70% of patients [6, 7]. Thus, there is an urgent requirement to identify effective diagnostic biomarkers for SONFH.

Circular RNAs (circRNAs) are a large class of noncoding RNAs. Advances in sequencing technologies have led to the recognition that circRNAs are well-expressed in peripheral blood. CircRNAs regulate gene expression by interacting with microRNAs (miRNAs) as sponges and participating in biological activities [8]. Recent studies have shown that circRNAs in blood samples are potential clinical biomarkers for various diseases [9, 10]. However, to our knowledge, no study has profiled circRNA expression in SONFH by RNA sequencing (RNA-seq).

To explore the functions of circRNAs in the development of SONFH, we undertook RNA-seq of samples from SONFH patients and healthy individuals. We identified differentially expressed circRNAs and predicted their functions and pathways. Putative networks of circRNA-miRNA-mRNA interactions were constructed for further studies on clinical diagnosis and treatment.

## 2. Materials and Methods

### 2.1. Case Information

The Institutional Review Board (Protocol Number: 2018-KL-075-02) approved the study, and every participant signed an informed consent form. Six peripheral blood samples were used for RNA-seq, including three patients with SONFH and three healthy control individuals with a history of GC use. Ten pairs for SONFH patients and control subjects were validated by qRT-PCR. The diagnosis of SONFH was based on each patient's history and imaging (radiograph, CT, and MRI). All patients in the case group were at Ficat stage II or III [11]. The inclusion criteria for SONFH and the control group were that they should have a history of GC use (>2 g within a 3-month period) and should have had no systemic disease, such as rheumatoid arthritis, ankylosing spondylitis, or systemic lupus erythematosus. The two groups were matched for age, sex, race, and region.

### 2.2. RNA Extraction and circRNA Sequencing

The HiPure PX Blood RNA Mini Kit (Amgen, Guangzhou, China) was used to extract total RNA from fresh blood mixed with three volumes of RNASafer LS Reagent (Amgen). The RNA concentration was obtained by a Qubit 3.0 fluorometer (Invitrogen, Carlsbad, CA, USA), and the integrity was evaluated with the Agilent 2100 Bioanalyzer (Applied Biosystems, Carlsbad, CA, USA). RNase R (Epicentre Technologies, Madison, WI, USA) was used to digest linear RNAs. CircRNA sequencing analysis was performed by Illumina HiSeq X Ten to identify circRNAs in patients with SONFH (*n* = 3) and control subjects (*n* = 3). CircRNAs showing a fold change > 1.5 and *p* < 0.05 were considered differential expressed. Raw sequence reads are presented in the Sequence Read Archive (SRA) database [12] (Number: PRJNA522627).

### 2.3. Bioinformatics Analysis

Functions of target genes and enrichment pathways were analyzed by using Gene Ontology (GO), Kyoto Encyclopedia of Genes and Genomes (KEGG), and Reactome analyses. CircRNA-miRNA-mRNA networks were predicted by miRanda.

### 2.4. qRT-PCR

qRT-PCR was performed to preliminarily confirm candidate circRNAs in 10 pairs of samples. Total RNA was extracted from all groups by using TRIzol reagent (Invitrogen) and RNeasy Plus Mini Kit (Qiagen, Guangzhou, China). qRT-PCR was implemented by using Geneseed qPCR SYBR Green Master Mix on an ABI 7500 system. The relative expression levels of selected circRNAs were determined using the 2^−△△CT^ method. Glyceraldehyde 3-phosphate dehydrogenase was utilized to normalize RNA preparations. Primer sequences for circRNAs were designed using Primer 5. The primer sequences of the eight selected circRNAs are shown in Table 1, and back-splicing sites are shown in Supplemental [Supplementary-material supplementary-material-1].

### 2.5. Statistical Analysis

All data were analyzed by SPSS 24.0 software. PCR data were analyzed by Student's *t*-test and presented as the mean ± standard error of the mean; *p* < 0.05 was considered statistically significant.

## 3. Results

### 3.1. CircRNA Identification

**Figure 1:**
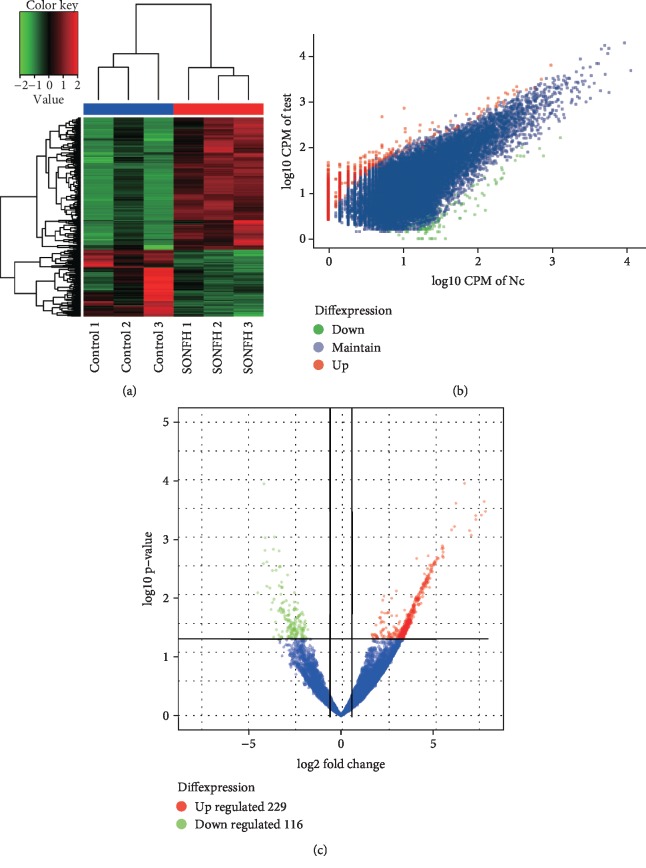
Circular RNA expression profile. (a) Hierarchical clustering for differentially expressed circRNAs. Red indicates upregulation; green indicates downregulation. (b, c) Scatter and volcano plots of the dysregulated circRNAs. Blue dots mean circRNAs with no statistical significance; red dots/green dots mean upregulated/downregulated circRNAs, respectively. SONFH: steroid-induced osteonecrosis of the femoral head.

RNA-seq was performed to profile circRNA expression from three patients with SONFH and three healthy individuals. A total of 33,098 circRNAs were discovered. We found that 229 circRNAs were upregulated, and 116 circRNAs were downregulated (Figure 1). Furthermore, we analyzed the category and distribution of the circRNAs (Supplemental [Supplementary-material supplementary-material-1]).

### 3.2. GO, KEGG, and Reactome Analysis

**Figure 2:**
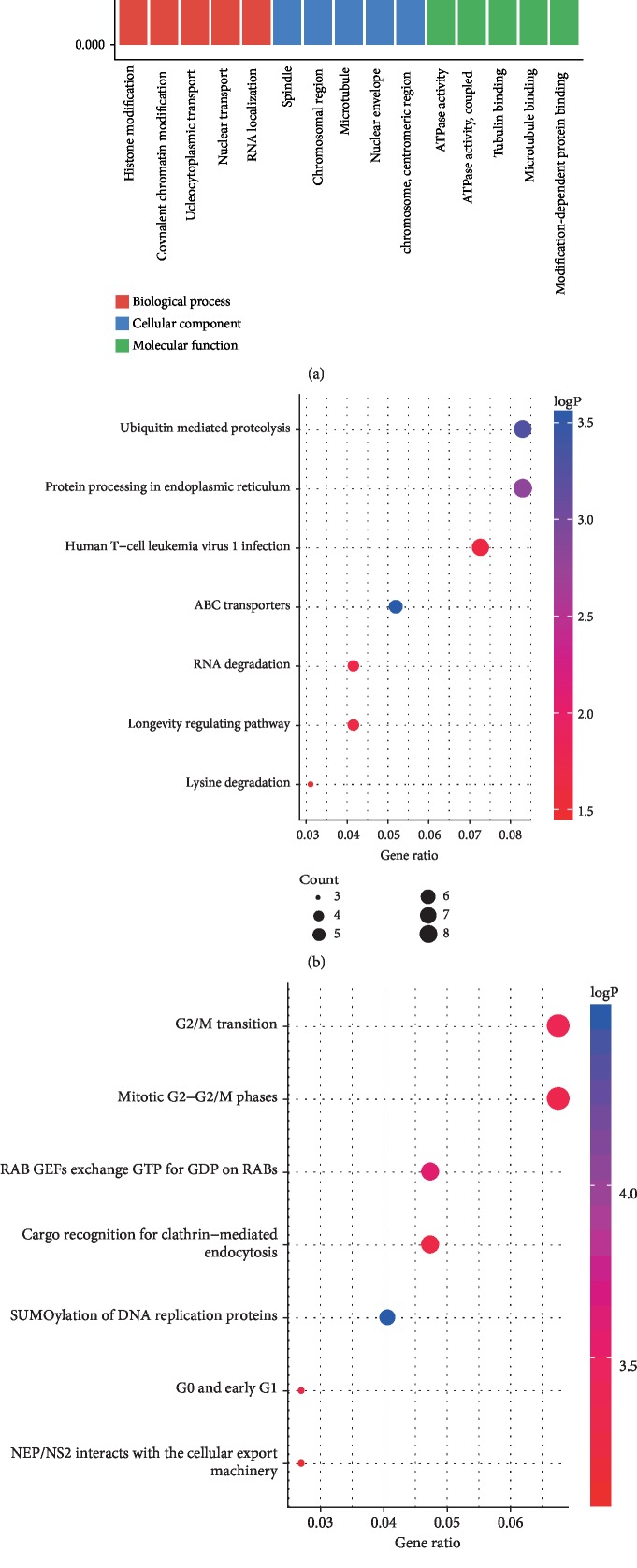
Function annotations for the differentially expressed circRNAs. (a) Gene ontology analysis is applied to clarify genetic regulatory networks in cellular component, biological process, and molecular function. (b) Top 7 enriched Encyclopedia of Genes and Genomes pathway. (c) Top 7 enriched Reactome pathway. A lower *p*-value indicates a higher significance of the pathway.

To infer the biological functions of the dysregulated circRNAs in the pathogenesis of SONFH, GO, KEGG, and Reactome analyses were used in this study to predict the function of circRNAs. GO analyses revealed that the most enriched GO terms were in cellular components, molecular functions, and biological processes. The most enriched KEGG pathway included ubiquitin-mediated proteolysis and protein processing in the endoplasmic reticulum. The top two pathways in the Reactome analysis were M phase and G2/M transition (Figure 2).

### 3.3. Validation of RNA-seq by qRT-PCR

**Figure 3:**
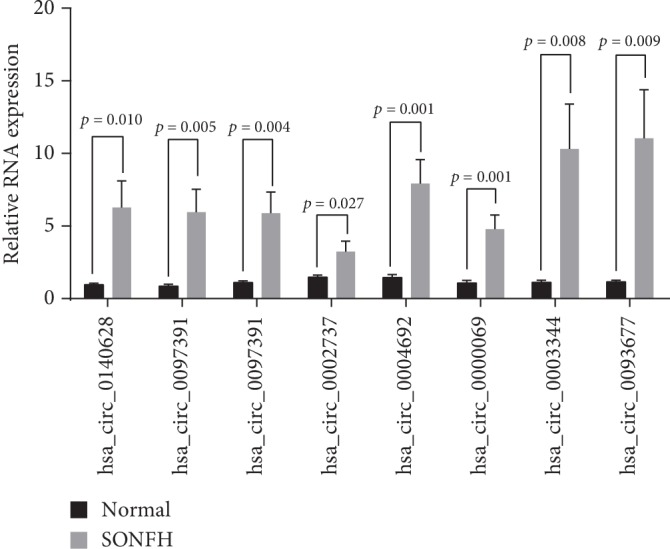
Preliminary validation of the eight selected circular RNAs. The relative levels of the eight circRNAs are confirmed by qRT-PCR. SONFH: steroid-induced osteonecrosis of the femoral head.

To verify the RNA-seq data, eight selected circRNAs were further validated by qRT-PCR. The results were consistent with the RNA-seq data (Figure 3).

#### 3.3.1. Prediction of circRNA-miRNA Interaction Networks and Target Genes for miRNAs

**Figure 4:**
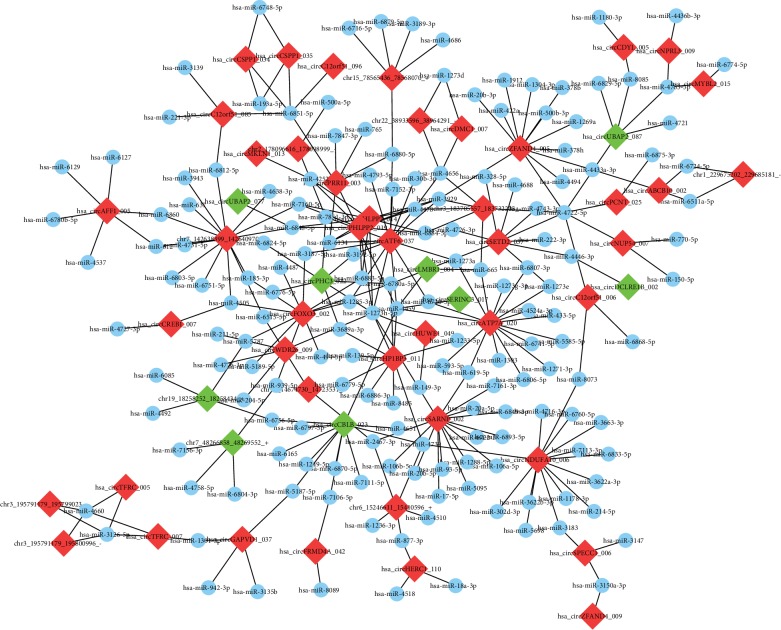
Prediction of circRNAs-miRNA networks. Software predicted high-binding potential miRNAs for each circRNA. The nodes with red and green colour represent upregulation and downregulation circRNAs, respectively, and nodes with blue colour represent miRNAs.

**Figure 5:**
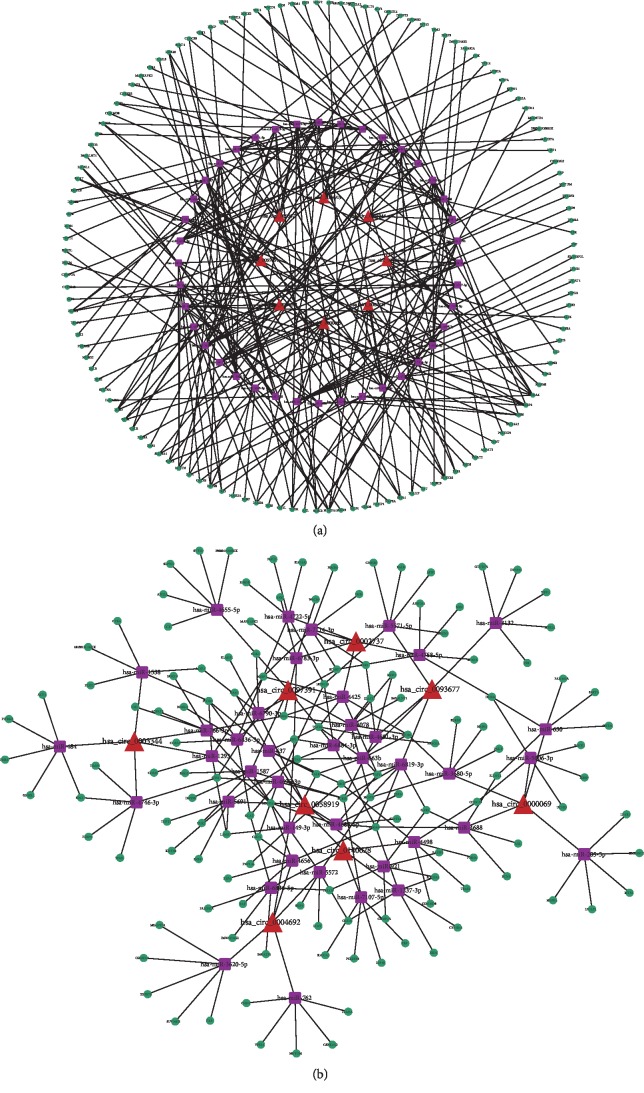
CircRNA-miRNA-mRNA network of the eight candidate circRNAs.

To explore the molecular mechanism and functions of the circRNAs, we investigated potential miRNAs binding with the circRNAs (Figure 4). The circRNA-miRNA-mRNA interaction network of the eight candidate circRNAs was predicted by miRanda and mapped by Cytoscape (Figure 5).

## 4. Discussion

In recent years, molecular biology has advanced considerably, and noncoding RNAs are attracting significant attention in the field of medicine. MiRNAs play important roles in the regulation of transcription [13]. Increasing studies have investigated differentially expressed miRNAs related to SONFH, identifying hundreds of such miRNAs [14]. For example, Li et al. investigated miRNA expression in SONFH patients and control individuals. Their data indicated that circulating miRNAs in the serum might play notable roles in the development of SONFH and act as diagnostic biomarkers [15]. Kao et al. compared miRNAs in peripheral blood by microarray and PCR, implying the possibility of using miRNAs as novel diagnostic or therapeutic targets [16].

Compared with miRNAs, circRNAs are more promising diagnostic biomarkers because they are more stable [17]. Being closed loops, circRNAs are not easily degraded by exonucleases. In addition, the half-life of circRNAs is doubled that of linear RNAs [18]. CircRNAs are reproducibly and easily detected in clinical blood samples. Stable and enriched circRNAs have been found in peripheral blood [19, 20], as well as blood components, such as exosomes [21], plasma [22], platelets [23], erythrocytes [24], and mononuclear cells [25].

For this study, we used peripheral blood. Our study found 229 upregulated circRNAs and 116 downregulated circRNAs. We further selected eight circRNAs for preliminary qRT-PCR validation based on a combination of previous studies and the circRNA-miRNA prediction network (Table 2) [15, 26–29]. The results indicated that the RNA-seq data was reliable and worthy of further study.

The use of GCs—oral, intravenous, or even inhaled—may alter the characteristics of peripheral blood and strongly affect the blood supply to the femoral head. Fat embolism and coagulation disorders are two of the most common causes of interrupted blood supply. GCs could directly or indirectly lead to hypofibrinolysis and thrombophilia, dysfunction and apoptosis of endothelial cells, lipid metabolism alterations, and platelet activation, which are followed by poor blood flow, ischemia, and eventually, osteonecrosis [30–33]. Nevertheless, the effectiveness of GCs differs among individuals owing to varied GC sensitivity [34].

As compared to invasive organ biopsy, the blood-based biomarker assay is a relatively economical and noninvasive method to detect disease, owing to its ease of accessibility and the low risk associated with sample collection. In previous studies, noncoding RNA expression patterns in peripheral blood have been used as the basis for the detection of disease [35–40]. However, peripheral blood can be affected by various factors. It is possible that changes in the expression profile of the disease reflect shifts in cell populations [41], which is a potential confounding factor to be considered.

Although we identified the differentially expressed circRNAs in SONFH, the underlying mechanism remains poorly understood. With the advance of high-throughput sequencing technologies, GO annotations have been good predictors of the functions and trends of genes [42]. KEGG is a reference knowledge database that describes the functional pathways that contribute to disease processes, and it is extensively used in enrichment analyses [43, 44]. Reactome analysis is utilized to visualize mRNA expression data and can be combined with other databases [45]. The molecular mechanism underlying the interactions of circRNAs and miRNAs in SONFH has not been deciphered. Therefore, the circRNA-miRNA-mRNA network was built based on our RNA-seq data. Understanding the interaction between the different pathways could provide novel strategies for managing bone disease [46]. These original findings might enhance our understanding of the functions of circRNAs in the mechanism of SONFH. For example, circ_0004692 may control hsa-miR-222-3p, which could further regulate the expression of the target gene SETD2. However, a single circRNA interacts with multiple miRNAs, and the target gene is regulated by many miRNAs. Network prediction may enrich future studies with novel perspectives to confirm the association between these dysregulated circRNAs and miRNAs in SONFH.

## 5. Conclusions

In conclusion, by RNA-seq, we identified 345 differentially expressed candidate circRNAs in SONFH. We also preliminarily confirmed the results by qRT-PCR with a small sample and conducted bioinformatics analysis to predict their functions. Our study provides a novel theoretical basis for further research on the functions of circRNAs in SONFH. However, large-sample validation and studies on specific regulatory mechanisms are required to realize the potential value of circRNAs as diagnostic biomarkers for SONFH in a more complete and in-depth manner.

## Figures and Tables

**Table 1:** Primer sequence list for the eight circRNAs for qPCR.

Gene	Primer sequences	PCR length (bp)
GAPDH	F 5′AGAAGGCTGGGGCTCATTTG3′	140
	R 5′GCAGGAGGCATTGCTGATGAT3′	

circ_0002737	F 5′TCAATCTCCAGAAAGCTTGCCG3′	133
	R 5′CATTTGCCTCCTCTTCAGCATG3′	

circ_0097391	F 5′CTTAGATGTTGATTCAGTTTTAG3′	147
	R 5′ATAGTCAATGTGTCTGACGCTT3′	

circ_0140628	F 5′AGCACAGTGATTGCTGGTTCCAT3′	139
	R 5′ACGTCATTCCCCTCACCTTTGAT3′	

circ_0003344	F 5′GATGCTCTGCTGGAAGAGCTA3′	151
	R 5′AGGTCTCCCTTATCGACCTGTA3′	

circ_0000069	F 5′AGACTACTTCAGGCACAGGTCT3′	164
	R 5′GTTCAGGAACTGATGGTTGGATC3′	

circ_0004692	F 5′ACCATGTGATCACAAGGCAGA3′	144
	R 5′GAATTCGATATACCTCCTTCATG3′	

circ_0058919	F 5′ATCAGTTCAGAGAGAGAGGA3′	145
	R 5′CCAGTACATATATTGCCATCTAC3′	

circ_0093677	F 5′GAATCTCAGCAAAAAGGTACAA3′	168
	R 5′TCATTGAAGAATGGAGGCTCT3′	

**Table 2:** The eight circRNAs were selected to perform further PCR validation.

hsa_circbase_ID	circBank_ID	Expression	hsa_mirbase_ID	Expression	FC	Gene
circ_0140628	circATP7A_020	Up	miR-433-5p [26]	Down [26]	12.707	ATP7A
circ_0097391	circC12orf51_085	Up	miR-221-3p [15]	Down [15]	11.190	HECTD4
circ_0058919	circNDUFA10_006	Up	miR-3663-3p [15]	Down [15]	10.913	NDUFA10
circ_0002737	circSARNP_002	Up	miR-17-5p [27]	Down [27]	13.144	SARNP
circ_0004692	circSETD2_057	Up	miR-222-3p [15]	Down [15]	12.420	SETD2
circ_0000069	circSTIL_025	Up	miR-345-5p [28]	Down [28]	4.401	STIL
circ_0003344	circTMCC2_001	Up	miR-615-5p [29]	Down [29]	9.783	TMCC2
circ_0093677	circZFAND4_005	Up	miR-378 h [27]	Down [27]	10.831	ZFAND4

## Data Availability

Raw sequence reads are presented in the Sequence Read Archive (SRA) database.

## Supplementary Materials

Supplementary 1 Supplementary figure 1. Back-splicing sites of the eight selected circRNAs.

Supplementary 2 Supplementary figure 2. Characteristic of the identified circular RNAs by RNA sequencing. (A) The pie diagram shows the genomic origin of the detected circRNAs. Most of the circRNAs originate from the exons. (B) The circos plots shows the dysregulated circRNAs on human chromosomes. CircRNA: circular RNA.
