# Supplementary figures and images for: Expression Profile Analysis of Differentially Expressed Circular RNAs in Steroid-Induced Osteonecrosis of the Femoral Head
Source: Dis Markers. 2019 Nov 15;2019:8759642. doi: 10.1155/2019/8759642 (PMC6885284; doi:10.1155/2019/8759642)

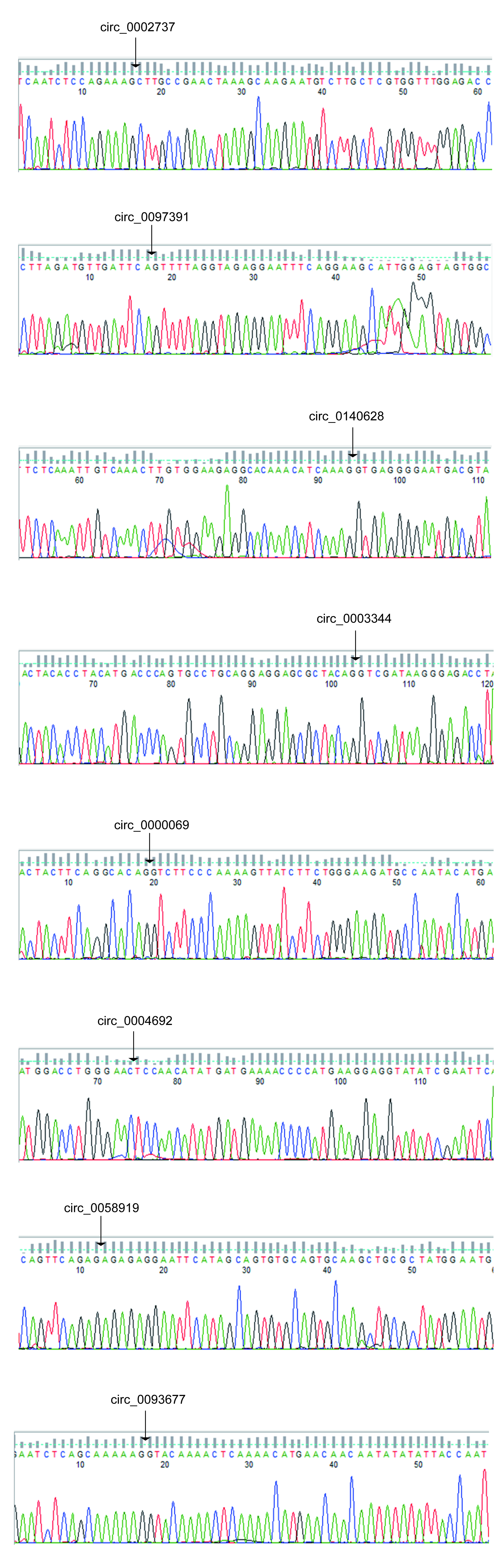

Supplement: Supplementary 1 — Supplementary figure 1. Back-splicing sites of the eight selected circRNAs. [file 8759642.f1.tif]

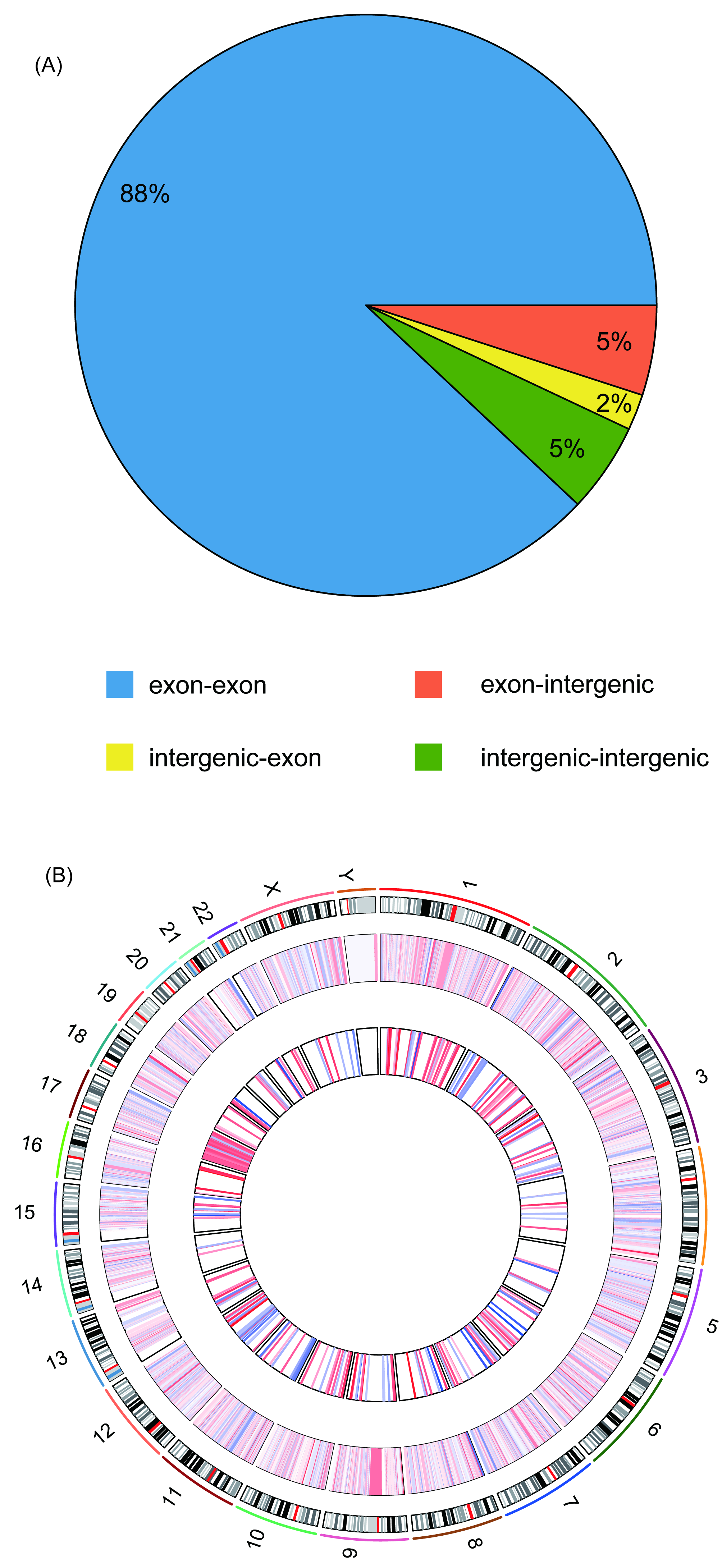

Supplement: Supplementary 2 — Supplementary figure 2. Characteristic of the identified circular RNAs by RNA sequencing. (A) The pie diagram shows the genomic origin of the detected circRNAs. Most of the circRNAs originate from the exons. (B) The circos plots shows the dysregulated circRNAs on human chromosomes. CircRNA: circular RNA. [file 8759642.f2.tif]
